# Risk of conversion from mild cognitive impairment to dementia in low‐ and middle‐income countries: A systematic review and meta‐analysis

**DOI:** 10.1002/trc2.12267

**Published:** 2022-03-13

**Authors:** Andrea M. McGrattan, Eduwin Pakpahan, Mario Siervo, Devi Mohan, Daniel D. Reidpath, Matthew Prina, Pascale Allotey, Yueping Zhu, Chen Shulin, Jennifer Yates, Stella‐Maria Paddick, Louise Robinson, Blossom C. M. Stephan, the DePEC team

**Affiliations:** ^1^ School of Biomedical, Nutritional and Sports Sciences, Faculty of Medical Sciences Newcastle University Newcastle upon Tyne UK; ^2^ Department Mathematics, Physics and Electrical Engineering Northumbria University Newcastle upon Tyne UK; ^3^ School of Life Sciences The University of Nottingham Medical School Nottingham UK; ^4^ Global Public Health Jeffrey Cheah School of Medicine and Health Sciences Monash University Malaysia Subang Jaya Malaysia; ^5^ International Centre for Diarrhoeal Disease Research, ICDDR,B Dhaka Bangladesh; ^6^ Department of Health Service and Population Research King's College London London UK; ^7^ Department of Psychology and Behavioural Sciences Zhejiang University Hangzhou China; ^8^ Institute of Mental Health Nottingham University Nottingham UK; ^9^ Translational and Clinical Research Institute Newcastle University Newcastle upon Tyne UK; ^10^ Gateshead NHS Community Health Foundation Trust Gateshead UK; ^11^ Population Health Sciences Institute Newcastle University Newcastle upon Tyne UK

**Keywords:** dementia, low‐ and middle‐income countries, mild cognitive impairment, risk factors

## Abstract

**Introduction:**

With no treatment for dementia, there is a need to identify high risk cases to focus preventive strategies, particularly in low‐ and middle‐income countries (LMICs) where the burden of dementia is greatest. We evaluated the risk of conversion from mild cognitive ompairment (MCI) to dementia in LMICs.

**Methods:**

Medline, Embase, PsycINFO, and Scopus were searched from inception until June 30, 2020. The search was restricted to observational studies, conducted in population‐based samples, with at least 1 year follow‐up. There was no restriction on the definition of MCI used as long as it was clearly defined. PROSPERO registration: CRD42019130958.

**Results:**

Ten thousand six hundred forty‐seven articles were screened; *n* = 11 retained. Of the 11 studies, most were conducted in China (*n* = 7 studies), with only two studies from countries classified as low income. A qualitative analysis of *n* = 11 studies showed that similar to high‐income countries the conversion rate to dementia from MCI was variable (range 6.0%–44.8%; average follow‐up 3.7 years [standard deviation = 1.2]). A meta‐analysis of studies using Petersen criteria (*n* = 6 studies), found a pooled conversion rate to Alzheimer's disease (AD) of 23.8% (95% confidence interval = 15.4%–33.4%); approximately one in four people with MCI were at risk of AD in LMICs (over 3.0–5.8 years follow‐up). Risk factors for conversion from MCI to dementia included demographic (e.g., age) and health (e.g., cardio‐metabolic disease) variables.

**Conclusions:**

MCI is associated with high, but variable, conversion to dementia in LMICs and may be influenced by demographic and health factors. There is a notable absence of data from low‐income settings and countries outside of China. This highlights the urgent need for research investment into aging and dementia in LMIC settings. Being able to identify those individuals with cognitive impairment who are at highest risk of dementia in LMICs is necessary for the development of risk reduction strategies that are contextualized to these unique settings.

## 1 INTRODUCTION

Mild cognitive impairment (MCI) defines an intermediate cognitive state between normal aging and dementia and is a target for dementia prevention and risk reduction research.^1^, ^2^ Numerous definitions for MCI exist and prevalence estimates vary (range < 1% to > 50%) depending on the population sampled (e.g., the age/sex distribution of participants, clinical‐based sample vs. individuals recruited from population‐based settings), MCI case definition, and operationalization of the component criterion for an MCI case diagnosis.^3^, ^4^, ^5^, ^6^, ^7^, ^8^ Further, within high‐income countries (HICs), the rates of conversion from MCI (across different subtypes) to dementia vary (range 10%–15% annually).^5^, ^6^, ^7^ Although some cases remain stable, others can revert to normal, with studies suggesting reversion ranges of 4% to 15% in clinic‐based samples^8^, ^9^, ^10^, ^11^ and 29% to 55% in population‐based samples.^12^, ^13^, ^14^, ^15^ In the absence of a cure for dementia, understanding the likelihood of, and risk factors associated with, conversion to dementia among MCI cases is important to help identify strategies for dementia risk reduction and prevention.^16^

The definition of MCI can be a difficult concept to disentangle. One of the most widely applied set of criteria, in clinical and research practice, are those defined by Petersen et al., describing patients with subjective memory loss verified by neuropsychological testing, with no significant impairment in other cognitive domains, no functional impairments, and no dementia.^17^ Other similar criteria have also been developed and applied including, for example, from the International Working Group,^18^ National Institute on Aging–Alzheimer's Association (NIA‐AA),^19^ and the American Psychiatric Association (Diagnostic and Statistical Manual of Mental Disorders, 4th Edition [DSM‐IV]).^20^ MCI is therefore an evolving concept, with varying definitions, which can make cross‐study comparisons challenging.^21^

While some have suggested that MCI as a method for classification of prodromal dementia can have a limited role in clinical and epidemiological settings, others argue that MCI could be a pragmatic tool for identifying individuals who could benefit from risk reduction.^22^ Modifiable risk factors for MCI and its conversion to dementia include health and lifestyle factors such as an unhealthy diet (e.g., a diet high in saturated fat, sugar, and salt), physical inactivity, smoking, cardiometabolic diseases (e.g., coronary heart disease and diabetes) and their risks including obesity and hypertension.^23^, ^30^ The literature on risk of conversion to dementia predominantly refers to MCI classified using Petersen criteria. ^24^ As discussed, with the multiple definitions of MCI available, this can make comparisons challenging. That said, this evidence highlights the potential for risk reduction and possible prevention or delay of dementia onset in MCI cases. Indeed, a recent report indicated that 21.7% of MCI cases that progress to dementia are potentially preventable, by targeting diet (using obesity as a proxy) (8.7%), diabetes (1.5%), and neuropsychiatric symptoms (11.5%).^23^

Compared to HICs, very few studies on MCI have been conducted in low‐ and middle‐income countries (LMICs).^4^ Extending the findings from a recent systematic review on MCI prevalence in LMICs,^4^ the aim of this systematic review and meta‐analysis was to identify and review longitudinal population‐based studies reporting on the risk of conversion from MCI to dementia in LMICs. The focus was on the rate of conversion and associated risk factors. Given that nearly two‐thirds of people with dementia live in LMICs^25^ identifying those individuals at highest risk is important for targeted interventions focused on reducing the burden of dementia in these settings.^26^

## 2 METHODS

This systematic review and meta‐analysis was conducted according to the PRISMA (Preferred Reporting Items for Systematic Reviews and Meta‐Analyses) guidelines (Appendix A.1 in supporting information).^27^ The study protocol was registered on the PROSPERO database (registration number CRD42019130958).

### 2.1 Search strategy and eligibility criteria

Four commonly used, comprehensive medical databases were searched electronically from inception until April 30, 2019. A second electronic database search was conducted from May 1, 2019 to June 30, 2020. The selected databases were: Medline; Embase; PsycINFO, assessed via Ovid (https://ovidsp.ovid.com); and Scopus (https://www.Scopus.com/home.uri). A detailed description of the search strategy is provided in the supporting information.

The search was restricted to observational studies, conducted in population‐based samples, with at least 1 year of follow‐up. Participants were those with a diagnosis of MCI according to internationally accepted and validated classifications.^1^, ^17^, ^18^, ^19^, ^28^, ^29^ Studies which included participants with “memory problems” or “self‐reported memory complaints” and no clear diagnosis of MCI were excluded. To be eligible, MCI participants had to be followed during the study period for risk of dementia; diagnosed according to established criteria, for example, National Institute of Neurological Disorders and Stroke–Alzheimer's Disease and Related Disorders Association (NINCDS‐ADRDA) criteria,^30^ DSM‐IV criteria,^20^, ^31^ or Neuro‐epidemiology Branch of the International Workshop of the National Institute of Neurological Disorders and Stroke with support from the Association Internationale pour la Recherche et l'Enseignement en Neurosciences (NINDS‐AIREN) criteria.^32^ All‐cause dementia and its subtypes (e.g., Alzheimer's disease [AD] and vascular dementia) were included. Studies that combined participants with MCI with another level of cognitive status at baseline (e.g., cognitively healthy or dementia), were only included if the MCI group data were analyzed and presented separately. Studies were excluded where the MCI sample was stratified by disease status (e.g., diabetes) and rates of conversion were not reported for the total population. Studies were required to be from a LMIC as per the Organisation for Economic Co‐operation and Development (OECD) criteria and World Bank classification, with inclusion based on the income status of the country at the time the study was conducted.

**Research in context**

1. Systematic Review: Medline, Embase, Scopus, and PsycINFO were searched for eligible articles reporting on conversion from mild cognitive impairment (MCI) to dementia in population‐ or community‐based studies from low and middle‐income countries (LMICs). The search strategy included terms that encompassed “dementia,” “mild cognitive impairment,” “incidence,” and “conversion.”
2. Interpretation: This is the first study to synthesize research on the risk of conversion to dementia in people with MCI in LMIC settings. We found that very few studies on risk of dementia in MCI cases have been undertaken in LMICs. Across the 11 studies, conversion to dementia was high and ranged from 6.0% to 44.8% over an average follow‐up of 3.7 years. A meta‐analysis of studies using Petersen criteria (*n* = 6 studies), found a pooled conversion rate to Alzheimer's disease (AD) of 23.8% (95% confidence interval = 15.4%–33.4%); approximately one in four people with MCI were at risk of AD in LMICs over 3.0 to 5.8 years follow‐up.
3. Future Directions: Like findings from high‐income countries, MCI is associated with increased risk of conversion to dementia in LMIC. There was, however, large heterogeneity in study methodology including definition of MCI, operationalization of MCI criterion, follow‐up time, and diagnosis of dementia as well as a scarcity of studies from countries classed as low income. There is an urgent need for LMICs to invest in the collection of robust, population‐based data, to determine the best strategies for identifying those individuals at highest dementia risk to inform the development of dementia risk reduction plans in these settings.

### 2.2 Screening process

**FIGURE 1:**
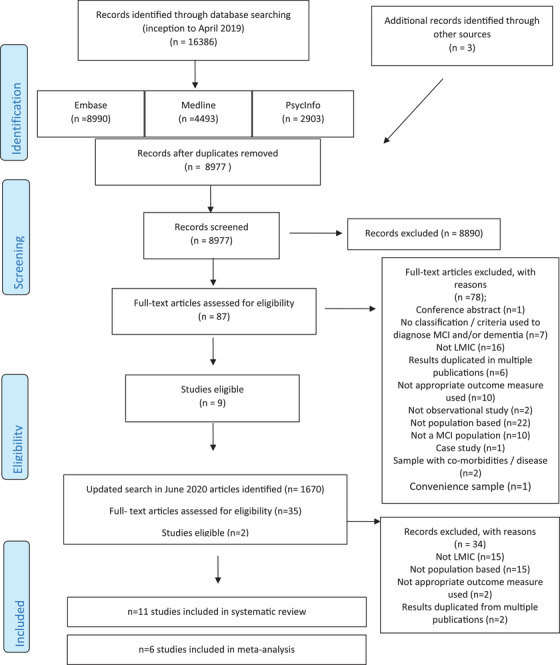
Study selection. LMIC, low‐ and middle‐income countries; MCI, mild cognitive impairment

Two reviewers independently assessed potentially relevant articles for eligibility (AMM and EP). The decision to include or exclude studies was hierarchical and initially made based on the study title and abstract to eliminate obviously irrelevant studies (see Figure 1). When a study's title/abstract could not be rejected with certainty, the full text of the article was obtained for evaluation. Discrepancies between reviewers were resolved by a third reviewer (BCMS). Next, full‐text articles were searched. In addition, the reference lists of all included articles were checked for any potentially missing papers.

### 2.3 Data extraction

A standardized form was used to extract data from the included studies for assessment of study quality and evidence synthesis. Extracted data included information on: (1) author and year of publication, (2) country, (3) age, (4) sample size, (5) follow‐up duration, (6) MCI and dementia diagnostic criteria, (7) analytical method for determining rate of conversion including loss to follow‐up, (8) results of conversion from MCI to dementia, and (9) risk factors for conversion to dementia (including details of all risk factors assessed—both significant and non‐significant). One author extracted data (EP), and a second checked the extraction (AMM). A third author (BCM) reviewed any discrepancies.

### 2.4 Risk of bias assessment

Quality assessment was guided by the Newcastle–Ottawa Scale for cohort studies.^33^ Risk of bias was assessed on three main categories: selection, comparability, and outcome. The maximum possible score was 8; three stars for selection, two stars for comparability, and two stars for outcome. Two authors (AMM and EP) independently assessed risk of bias, with any disagreement resolved by discussion with a third assessor if required.

### 2.5 Analysis

For each study, we report the proportion of MCI cases that converted to dementia for each definition of MCI separately. This was calculated as the ratio of those who converted to dementia over the total sample size. We also report on the key risk factors significantly associated with an increased risk of dementia. Details of all risk factors assessed are in Table S2 in supporting information.

Where there were multiple studies using the same criteria to diagnosis MCI and dementia, a meta‐analysis was undertaken. This was only possible for studies that diagnosed MCI using Petersen‐type criteria with an outcome of AD (*n* = 6 studies). The analysis was run in Stata using the Metaprop command to compute the meta‐analysis of pooled proportions. This allows computation of 95% confidence intervals (95% CI) using the score statistic and the exact binomial method and incorporates the Freeman‐Tukey double arcsine transformation of proportions to compute the weighted pooled estimate for normality assumptions. The program also allows the within‐study variability to be modeled using the binomial distribution.^34^ Given large differences in the design and sampling across studies, the random effects model was computed. Heterogeneity was assessed using the I^2^ statistic.

## 3 RESULTS

### 3.1 Study selection

From the electronic search, the titles and abstracts of 8977 publications were screened, and the full texts of 87 articles reviewed. The screening and study selection process is illustrated in the PRISMA flow diagram (Figure 1). Nine articles met the eligibility criteria. The most common reasons for exclusion were that the study was not from a LMIC, the sample was not population‐based, and the study did not report incident dementia. A second search conducted in June 2020 identified a further 1670 articles, from which two studies were eligible for inclusion. Thus, 11 articles are included in this review.

### 3.2 Study characteristics

Of the 11 studies, most were conducted in China (*n* = 7 studies),^35^, ^36^, ^37^, ^38^, ^39^, ^40^, ^41^ followed by Brazil (*n* = 2 studies),^42^, ^43^ Nigeria (*n* = 1 study^44^),^44^ and Tanzania (*n* = 1 study).^45^ Only one study was nationally representative using census data from Rio Grande do Sul, Brazil.^42^ At the time of participant recruitment, two studies^44^, ^45^ were from low‐income countries while the majority (*n* = 9 studies)^35^, ^36^, ^37^, ^38^, ^39^, ^40^, ^41^, ^42^, ^43^ were from middle‐income countries. MCI sample size at baseline ranged from *n* = 21^42^ to *n* = 837,^35^ with a mean of *n* = 370 (standard deviation [SD] = 295). Most studies included participants aged > 60 years (*n* = 6 studies),^37^, ^38^, ^39^, ^40^, ^41^, ^42^ while others included those > 55 years (*n* = 2 studies),^35^, ^36^ > 65 years (*n* = 1 study),^44^ and > 70 years (*n* = 2 studies).^43^, ^45^ Duration of follow‐up ranged from 2 years^44^ to 5.8 years,^42^ with a mean of 3.7 years (SD = 1.1 year). At follow‐up, MCI sample size ranged from *n* = 21^42^ to *n* = 638,^35^ with a mean of *n* = 298 (SD = 238).

### 3.3 Diagnostic criteria for MCI

Most studies diagnosed MCI using Petersen criteria^17^, ^28^, ^46^, ^47^ (*n* = 5 studies).^35^, ^36^, ^38^, ^40^, ^41^ Other criteria included the Clinical Dementia Rating scale (CDR 0.5;^48^ *n* = 1 study),^43^ Dubois 2004 criteria^49^ (*n* = 1 study), ^42^ the criteria from the DSM‐IV^31^ (*n* = 2 studies),^37^, ^39^ and the International Working Group on MCI criteria^18^ (*n* = 1 study).^45^ Only 1 study^44^ used the Cognitive Impairment No Dementia (CIND) classification.^50^ This paper^44^ reports that of the *n* = 87 CIND participants, *n* = 74 were classed as having “medically unexplained memory loss” (MUML) described as comparable to MCI using Petersen criteria.^51^ For this review, the total sample of *n* = 87 CIND participants were included. Table 1 shows a description of the MCI diagnostic criteria used across the different studies. The majority of studies classified participants as MCI (*n* = 9 studies),^35^, ^36^, ^37^, ^38^, ^40^, ^41^, ^42^, ^43^, ^45^ while others subtyped MCI into amnestic MCI (aMCI; *n* = 2 studies), ^39^, ^41^ aMCI single domain (*n* = 1 study),^41^ aMCI multiple domain (*n* = 1 study),^41^ non‐amnestic MCI single domain (*n* = 1 study),^41^ and non‐amnestic MCI multiple domain (*n* = 1 study).^41^

**TABLE 1:** Study characteristics (grouped by criteria for MCI diagnosis and ordered by age)

				Sample size						
Reference	Country	Population characteristics	Age (years)	Total sample at baseline	MCI sample	MCI sample at follow up	Mean Follow‐up (years)	MCI Type	Dementia criteria	Conversion to dementia
Petersen criteria^17^, ^28^, ^47^, ^81^ (*n* = 5 studies)										
Huang et al.^36^	China	29 geographically defined communities located within Greater Beijing, China (12 urban and 17 rural)	>55	5743	175	121	3	MCI	DSM‐IV (all cause) NINCDS‐ADRDA (AD) NINDS‐AIREN (VaD)	42% (51/121 including *n* = 29 with AD, *n* = 18 with VaD and *n* = 4 with other dementias (after 3 years follow up)
Li et al.^35^	China	10 randomly selected communities in the city of Chongqing	>55	26,481 (18,683 screened)	837	638	5	MCI	DSM‐IV (all cause) NINCDS/ADRDA (AD)	44·8% (298/638 dementia including *n* = 298 AD) (after 5 years follow up)
Ding et al.^41^	China	Jingansi community in downtown Shanghai, China	>60	3141	655	362	4	MCI aMCI aMCI‐SD aMCI‐MD naMCI‐SD naMCI‐MD	DSM‐IV (all cause)	**MCI** 6·0 (95% CI: 4·7–7·3) per 100 person‐years (79/362) **aMCI** 6·9 (95% CI 5·2–8·6) per 100 person years (59/238) **aMCI‐SD** 3·0 (95% CI: 1·6–4·4) per 100 person‐years (17/156) **aMCI‐MD** 14·2 (95% CI: 10·2–18·2) per 100 person years (42/82) **naMCI‐SD** 2·7 (95% CI: 1·0–4·5) per 100 person‐years (9/91) **naMCI‐MD** 8·7 (95% CI: 3·8–13·7) per 100 person‐years (11/33) (after 4 years follow up)
Yang et al.^40^	China	Chinese community dwelling elder people	>60	652	465	465	3	MCI	DSM‐ IV‐TR (AD)	16·8% (78/465) (after 3 year follow up)
Yu et al.^38^	China	8 geographically convenient communities in Taiyuan city, China	>60	6192	600	518	5	MCI	NINCDS‐ADRDA (AD)	17·0% (89/518) (after 5 years follow up)
CIND based on Levy and Working Party of the International Psychogeriatric Association in collaboration with the WHO^50^ (*n* = 1 study)										
Baiyewu et al.^44^	Nigeria	Idikan community and adjacent wards of the city of Ibadan, Nigeria	>65	2487	152	87	2	CIND	DSM III‐R ICD‐10 (all cause)	16·1% (14/87) developed dementia (after 2 years follow up)
CDR^82^ (*n* = 1 study)										
Montano et al.^43^	Brazil	A community cohort living in an urban district, in São Paulo city	>70	1667	80	40	2.6	CDR 0.5	NINCDS–ADRDA (all cause)	37·5% (145.4 per 1,000 person‐years; 15/40) (after follow up)
IWG^18^ (*n* = 1 study)										
Paddick et al.^45^	Tanzania	6 randomly selected villages from the Hai District in Northern Tanzania.	>70	296	46	46	4	MCI	DSM‐IV (all cause) NINCDS‐ADRDA (AD) NINDS‐ AIREN (VaD)	37·0% (17/46) all cause (*n* = 9 AD, *n* = 4 VaD, *n* = 2 Parkinson disease dementia & *n* = 2 mixed aetiology) (after 4 years follow up)
DSM‐IV Criteria^31^ (*n* = 2 studies)										
Yu et al.^37^	China	26 military cadres’ sanatoriums of Shijiazhuang city	>60	2674	216	209	3	MCI	DSM‐IV (all cause) NINCDS‐ADRDA (AD)	24·4% (51/209) all cause 15·7% (35/209) AD (per year)
Wang et al.^39^	China	9 densely distributed elderly communities in Taiyuan	>65	6152	600	557	3	aMCI	NINCDS‐ADRDA (AD)	6% (34/557 per year)
Dubois and Albert, 2004^49^ (*n* = 1 study)										
Godhino et al.^42^	Brazil	Older community dwelling residents in the catchment area of the Hospital de Clinicas de Porto Alegre, Rio Grande do Sul, Brazil	>60	245	21	21	5.8	MCI	DSM‐IV & NINCDS‐ADRDA (AD)	38·1% (8/21) (after 5.8 years follow up)

Abbreviations: AD, Alzheimer's disease; aMCI, amnestic mild cognitive impairment; CIND, cognitive impairment no dementia; CDR, Clinical Dementia Rating; DSM‐IV, Diagnostic and Statistical Manual of Mental Disorders Fourth Edition; ICD, International Classification of Diseases; IWG, International Working Group; MCI, mild cognitive impairment; NIA‐AA, National Institute on Aging and Alzheimer's Association; NINCDS‐ADRDA, National Institute of Neurological Disorders and Stroke‐Alzheimer's Disease and Related Disorders Association; NINDS‐AIREN, National Institute of Neurological Disorders and Stroke International Workshop with support from the Association Internationale pour la Recherche et l'Enseignement en Neurosciences; VaD, vascular dementia; WHO, World Health Organization.

### 3.4 Diagnostic criteria for dementia

Most studies (*n* = 7 studies)^35^, ^36^, ^37^, ^40^, ^41^, ^42^, ^45^ defined incident dementia using the DSM‐IV criteria.^20^, ^31^ This was followed by NINCDS‐ADRDA criteria^49^, ^52^ (*n* = 5 studies),^35^, ^36^, ^37^, ^38^, ^39^ the NINDS‐AIREN criteria^32^ (*n* = 2 studies),^35^, ^36^ and DSM‐III‐R criteria^20^ (*n* = 1 study^44^).^44^ Studies defined dementia as either all‐cause (*n* = 7 studies),^35^, ^36^, ^37^, ^41^, ^43^, ^44^, ^45^ AD (*n* = 8 studies),^35^, ^36^, ^37^, ^38^, ^39^, ^40^, ^42^, ^45^ or vascular dementia (VaD; *n* = 2 studies). ^36^, ^45^ One study^44^ mentioned using International Classification of Diseases (ICD)‐10 criteria from the World Health Organization.^53^

### 3.5 Conversion from MCI to dementia

Rates of conversion from MCI to dementia ranged from 6.0%^39^ to 44.8%^35^ for all‐cause dementia (*n* = 11 studies) over an average follow‐up of 3.7 years (SD 1.2 years); 6.0%^35^, ^39^ to 46.7%^25^ for AD (*n* = 8 studies) over an average follow‐up of 4.0 years (SD 1.1 years), and 8.7%^45^ to 14.9%^36^ for VaD (*n* = 2 studies) over an average follow‐up of 3.5 years (SD 0.7 years). Rates of conversion to all‐cause dementia were generally higher for those definitions that captured broader impairment; for example, range of conversion for all‐MCI (*n* = 8 studies),^35^, ^36^, ^37^, ^38^, ^40^, ^41^, ^42^, ^45^ CIND (*n* = 1 study),^44^ and CDR (*n* = 1 study)^43^ (range 16.8% to 44.8% over 2.0–5.8 years follow‐up) compared to more restricted definitions of single domain MCI; for example, aMCI range 6.0% to 6.9% over an average of 3.5 years follow‐up (*n* = 2 studies).^39^, ^41^

### 3.6 Risk factors for dementia

Risk factors for MCI conversion to dementia were investigated in 10 (out of 11) studies.^35^, ^36^, ^38^, ^39^, ^40^, ^41^, ^42^, ^43^, ^44^, ^45^ Significant risks included older age (*n* = 6/10 studies^19^, ^35^, ^38^, ^39^, ^40^, ^41^, ^44^), poor baseline performance on cognitive tests (*n* = 5/7 studies^36^, ^40^, ^41^, ^42^, ^43^), sex (being female; *n* = 3/10 studies^35^, ^38^, ^44^), hypertension (*n* = 3/6 studies^35^, ^38^, ^39^), low educational attainment (illiteracy or primary school; *n* = 2/10 studies^35^, ^36^), anxiety and depression (*n* = 2/5 studies^39^, ^40^), history of stroke (*n* = 1/4 studies^36^), diabetes (*n* = 2/4 studies^35^, ^39^), and apolipoprotein E (*APOE*) ε4 status (*n* = 2/4 studies^35^, ^39^). Full details of all risk factors assessed in each study are presented in Table S2.

### 3.7 Risk of bias assessment

Full details of the risk of bias assessment can be found in Table S3 in supporting information. The included studies averaged 7.3 stars out of 10 (range 5–8). Eight studies (out of 11) scored the maximum of eight stars.

### 3.8 Meta‐analysis—risk of conversion to AD

**FIGURE 2:**
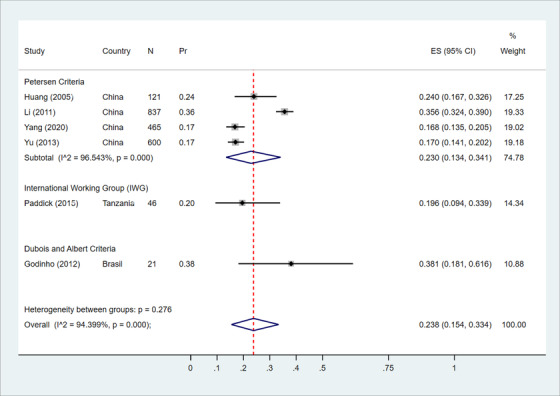
Forest plot showing the meta‐analysis of the proportion of the mild cognitive impairment converting to Alzheimer's disease. CI, confidence interval; Effect Size (ES), xxxxxxxxx

Six studies were included in the meta‐analysis; *n* = 4 from China^35^, ^36^, ^38^, ^40^ and *n* = 1 each from Tanzania^45^ and Brazil.^42^ Across the six studies, MCI sample size ranged from *n* = 21^42^ to *n* = 837^35^ and age from > 55 years^35^, ^36^ to > 70 years.^45^ Figure 2 presents the study‐specific proportion of Petersen‐defined MCI cases that converted to AD over time with 95% CIs for each study as well as the Chinese subgroup and overall pooled estimate with 95% Wald confidence interval and the I^2^ statistics. As shown, significant intra‐group heterogeneity among China‐based studies was observed (*P* < .0001 with I^2 ^= 96.54%). When pooling the six studies together conversion to dementia was estimated at 23..8% (95% CI = 15.4%–33.4%) over a range of 3.0–5.8 years.

## 4 DISCUSSION

In this systematic review and meta‐analysis, we found that very few studies on risk of MCI conversion to dementia have been undertaken in LMIC settings. This contrasts to the significant research investment into MCI (and more specially dementia) in HICs. Across the 11 studies, conversion to all‐cause dementia (including all dementia, AD, and VaD) was high and varied, ranging from 6.0% to 44.8% over an average follow‐up of 3,7 years. Similar variability in risk of conversion to dementia has been reported in studies from HICs.^5^, ^6^, ^7^ For studies using Petersen criteria (*n* = 6), the pooled conversion rate to AD was 23.8% over 3.0 to 5.8 years. This suggesting that approximately one in four people with MCI are at risk of developing AD in LMICs.

While risk of dementia in MCI has been studied extensively in HICs, in contrast very little research has been undertaken in LMIC settings. Further, the majority of studies were from China (middle‐income) with data from only three other countries represented including Brazil^42^, ^43^ (upper middle income), Nigeria^44^ (lower middle income), and Tanzania^45^ (low income). Limited resources for research, including access to funding, and infrastructure to diagnose MCI/dementia in LMIC settings could be possible reasons for this scarcity. However, this is a significant research gap particularly considering the high burden of cognitive impairment and dementia in low‐income country settings.^25^ Of the studies included in this review, there was also variability in terms of outcome measure (all‐cause dementia vs. AD vs. VaD), length of follow‐up, sample size, and diagnostic criteria for MCI. In addition, the examination of MCI conversion to other dementia subtypes was limited as only AD and VaD were investigated. Therefore, there is an urgent need for future studies to attempt to standardize the methodology used to allow for better cross‐study comparisons, aiming for studies to be population representative and generalizable.

Studies from HICs estimate annual conversion from MCI (irrespective of MCI definition used) to dementia at approximately 3% to 10% in community settings and 10% to 15% in clinical settings.^5^, ^16^, ^17^, ^18^, ^54^ Similar to findings from HCIs, conversion rates were found to be variable across the different LMICs sites ranging from 6.0% to 44.8%. Rates of conversion to dementia were generally higher for those definitions that capture broader impairment (e.g., range of conversion for all‐MCI, CIND, and CDR: range 16.8% to 44.8% over 2.0–5.8 years follow‐up) compared to more restricted definitions of single domain MCI (e.g., aMCI range 6.0% to 6.9% over an average of 3.5 years follow‐up).^55^, ^56^, ^57^, ^58^, ^59^, ^60^, ^61^ Given the high reported prevalence of MCI in LMIC settings^4^ in addition to the high dementia conversion rates reported here, the development of strategies to prevent or delay dementia progression in those individuals with cognitive impairment could have a significant impact on the burden of disease associated with mental health conditions in these settings.

Similar to findings from HICs, non‐modifiable risk factors for progression to dementia from MCI included age^62^, ^63^, ^64^, ^65^ and *APOE* ε4 allele status.^51^, ^66^, ^67^ Regarding sex, while being female has been found to be associated with increased risk of prevalent MCI,^4^ and has been associated with higher risk of progression to dementia in HICs,^68^ only 2 studies^38^, ^44^ out of 10 that investigated sex effects found that being female was a risk factor for conversion from MCI to dementia. Research evidence, predominantly from HICs, suggests a putative link between sex and/or educational attainment and cognition.^69^, ^70^, ^71^, ^72^ However, methodological weaknesses and potential of reverse causality within these studies adds limitations to their interpretation and warrants longitudinal studies with longer follow‐up.^71^ Furthermore, key modifiable risk factors were also similar to those reported in HIC settings, including poor cardiometabolic health, the presence of vascular risk factors, and poor neuro‐psychiatric health such as the presence of depression.^23^, ^62^, ^73^ Targeting these factors could be an early strategy for not only preventing MCI, but also reducing the burden of dementia. Research evidence suggests that up to 40% of dementia cases may be preventable through targeting 12 modifiable risk factors,^74^ many of which can be influenced by diet and lifestyle practices.^75^, ^76^ Emerging evidence also indicates that non‐pharmacological interventions such as cognitive training may reduce dementia risk.^77^ Further work is required to identify if these strategies are plausible for those with MCI, and feasible within LMIC settings.

This is the first study to synthesize research on the risk of dementia in people with MCI in LMIC settings. We undertook a wide literature search capturing many of the different definitions of MCI and included studies of all‐cause dementia and its subtypes. While there was variability in how dementia was diagnosed, most (63.6%) studies used DMS‐IV criteria. However, there are some limitations. The electronic search was undertaken in English and therefore studies published in other languages, including those common in LMICs such as Spanish, Portuguese, and French could have been missed. Although we used a wide search strategy to ensure that we captured all studies on the topic, we did not search the gray literature, which could have resulted in missing non‐published studies highlighting a risk of publication bias. Only a limited number of studies were identified and only one study was from a nationally representative sample.^42^ This makes it hard to generalize the results especially across different LMIC settings particularly LMIC countries in Eastern Europe, the Middle East, and Global South where no data are currently available. While most studies were associated with low risk of bias, there was variability in study robustness, for example, in terms of MCI diagnosis (including cognitive test used), sample size, and participant selection. While all studies were population‐ or community‐based, there was large heterogeneity in study methodology including definition of MCI, operationalization of MCI criterion, follow‐up time, reporting of conversion rates (annually vs. after number of years of follow‐up), and diagnosis of dementia, all of which could impact the results. In addition, due to the small number of studies included it was not possible to stratify the meta‐analysis by age or follow‐up duration. Last, we should emphasize that the studies in the meta‐analysis are limited to a few studies from three countries only, with different characteristics and profiles of the population. Indeed, heterogeneity was high and possibly reflects differences in sample selection, sample characteristics (age and sex distribution), sample size, and operationalization of MCI criteria (Figure S1 in supporting information). The results support calls for an urgent need to harmonize methodology in MCI and dementia research to improve cross‐study comparability.^78^ Indeed, future studies could draw on current recommendations^78^ for harmonization in the methods of conducting dementia and MCI work and intelligent data synthesis in HICs as well as specifically in LMICs including the 10/66 Study protocol^79^ and the Harmonized Cognitive Assessment Protocol (HCAP),^80^ all of which outline recommendations for cognitive assessment tools and interviewing methods to improve cross‐study comparability. Until there is agreed‐upon methodology for MCI/dementia research globally, evidence synthesis findings, such as the findings here, should be interpreted with caution.

There is an urgent need for research investment into robust, population‐representative studies focused on risk of cognitive impairment and dementia in LMICs using harmonized methodology.^78^ This is necessary to make it possible to campaign for prioritization of funding toward cognitive screening and risk reduction. This would also allow investment in better education and development of infrastructure in these settings to improve knowledge of diagnosis and risk factor management, but also facilitate the implementation of more population representative, robust studies, particularly in countries of low income.

Two‐thirds of people with dementia live in LMICs, where resources, services, research, and support for older age care are limited and often non‐existent. While dementia is currently incurable, results from HICs suggest that early interventions focused on reducing risk could lessen the number of people who develop dementia in the future. This would result in major health benefits and reduced public spending. As highlighted by this review little comparative data on MCI exists in LMIC settings. The results suggest that MCI is associated with risk of conversion to dementia in LMICs and may be influenced by demographic (e.g., age) and health (e.g., cardiometabolic disease) factors, but more research is needed particularly in low‐income settings.

## CONFLICTS OF INTEREST

Andrea M. McGrattan, Eduwin Pakpahan, Mario Siervo, Daniel D. Reidpath, Matthew Prina, Yueping Zhu, Chen Shulin, and Louise Robinson report no relevant disclosures. Authors who have received grants or contracts from any entity in the past 36 months: Devi Mohan—(1) Dementia toolkit for carers (DeToC): A comprehensive toolkit to support carers of people living with dementia in rural communities. Monash University Malaysia ASEAN grant; Role: Principal investigator. (2) Salivary lactoferrin for early identification of sleep related cognitive decline: A potential target for dementia prevention. Monash University Malaysia, School Strategic Grant; Role: Principal investigator. (3) Pathways of Acquired Epilepsy and their Comorbidities: A Translational Crosstalk between HMGB1 Mechanisms and Gut Microbiome. Monash University Malaysia, School Strategic Grant; Role: Co‐Investigator. (4) Global dementias: Examining structural vulnerability and dementia outcomes. Australian Research Council: Discovery Projects; Role: Partner investigator. (5) Investigating Multimorbidity Through capacity building (MUTUAL). MRC UK‐ GCRF Global Multimorbidity—Seed Funding; Role: Co‐Investigator. (6) Improving early detection and diagnosis of breast cancer among multi‐ethnic rural communities in Malaysia—the implementation of the community education and navigation programme (CENP). Newton Fund Impact Scheme Grant; Role: Co‐investigator. (7) Dementia Prevention and Enhanced Care (DePEC). National Institute of Health Research, UK: Global Health Group (Dementia)‐ (16/137/62); Role: Co‐investigator (all payments made to the institution). Jennifer Yates—NIHR RfPB Co‐investigator Enhance project (all payments made to the institution). Stella‐Maria Paddick—British council science South Asia grant; Broadening our horizons scheme; GCRF funding (Leicester university) for biomarker analysis; all small grants competitively awarded for research costs (all payments made to the institution). All authors who have received funding to travel to attend meetings in the past 36 months: Stella‐Maria Paddick—Cumbria, Northumberland, Tyne and wear NHS foundation trust—travel to Dhaka for a work related meeting; Newcastle University broadening our horizons scheme 2018—reciprocal visit between UK and Chile for one researcher at each site (all payments made to the institution). Blossom C. M. Stephan—NIHR Funded Global Health Research Group on Dementia Prevention and Enhanced care to attend ADI2020 conference (virtual due to COVID‐19; all payments made to the institution). All authors who have been members of external committees (within the past 36 months): Blossom C. M. Stephan—member of the Alzheimer's Society Research Strategy Council (chair the Dementia Prevention Subcommittee); an invited participant to the World Dementia Council, Prevention Workshop, 2021. Stella‐Maria Paddick—on the executive committee of the Royal College of Psychiatrists International Psychiatry and Volunteering Special interest group and attended a number of online meetings. Other financial or non‐financial interests in the past 36 months: Stella‐Maria Paddick—employed by the UK National Health Service.

## AUTHOR CONTRIBUTIONS

Blossom C. M. Stephan conceptualized the study. Andrea M. McGrattan and Eduwin Pakpahan undertook screening and data extraction. Blossom C. M. Stephan acted as third reviewer. Eduwin Pakpahan undertook the meta‐analysis. Andrea M. McGrattan drafted the initial manuscript, and Blossom C. M. Stephan and Eduwin Pakpahan critically reviewed and edited. All authors had full access to all the data in the study and had final responsibility for the decision to submit for publication.

## Supporting information



SUPPORTING INFORMATION

SUPPORTING INFORMATION

## Data Availability

This systematic review was registered via Prospero; PROSPERO registration number: CRD42019130958. A copy of the systematic review protocol is available on request and can be provided by the corresponding author. Requests for access to the data reported in this article will be considered by the corresponding author.
