# Supplementary figures and images for: Risk of conversion from mild cognitive impairment to dementia in low‐ and middle‐income countries: A systematic review and meta‐analysis
Source: Alzheimers Dement (N Y). 2022 Mar 13;8(1):e12267. doi: 10.1002/trc2.12267 (PMC8918697; doi:10.1002/trc2.12267)

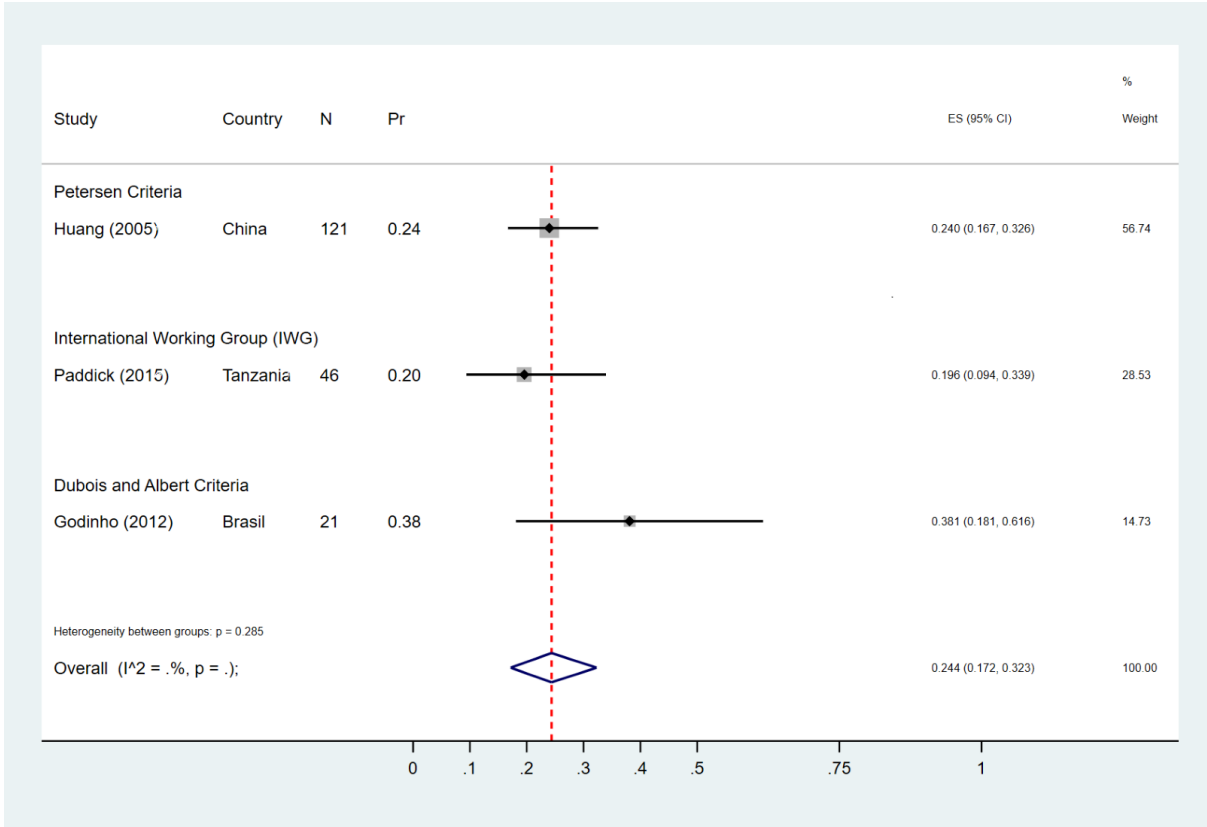

**Supplementary Material: Figure 1: Sensitivity analysis of Huang (2005), Paddick (2015) and Godinho (2012)**

Supplement: Supplementary file 1 — SUPPORTING INFORMATION [file TRC2-8-e12267-s001.pdf]
