# Supplementary material for: Risk of conversion from mild cognitive impairment to dementia in low‐ and middle‐income countries: A systematic review and meta‐analysis
Source: Alzheimers Dement (N Y). 2022 Mar 13;8(1):e12267. doi: 10.1002/trc2.12267 (PMC8918697; doi:10.1002/trc2.12267)
Supplement: Supplementary file 2 — SUPPORTING INFORMATION [file TRC2-8-e12267-s002.docx]

**Supplementary Material File:**

| **Supplementary Material Table 1 Search Strategy – Mesh terms for Medline OVID search (Adapted per database)** |
| --- |
| ("age related cognitive decline" OR "age associated memory impairment" OR "amnestic mild cognitive impairment" OR "multiple mild cognitive impairment" OR "mild dementia" OR "benign senescent forgetfulness" OR "subjective memory complaint*" OR "questionable dementia" OR "age associated cognitive decline" OR "cognitive impairment no dementia" OR "minimal dementia" OR "mild cognitive disorder*" OR "limited cognitive disturbance*" OR "mild neurocognitive disorder*" OR "mci due to Alzheimer's disease" OR "non-amnestic mild cognitive impairment" OR “MMSE” OR “ADAS” OR "cdr 0.5 " OR "mild cognitive impairment")  AND (dementia incidence OR dementia inc$ OR incident dementia OR inc$ dementia OR dement* OR Alzheimers disease incidence OR Alzheimers disease inc$ OR alzheimer* OR Lewy body dementia incidence OR Lewy body dementia inc$ OR lewy* adj2 bod* OR parkinson* disease dementia incidence OR parkinson* disease dementia inc$ OR parkinson* disease dementia OR progression OR conversion) |

**Supplementary Material Table 2** **Risk factors for MCI conversion to dementia for each study**

| Reference | Country | Type of analysis | Risk factors tested | Statistically significant risk factors |
| --- | --- | --- | --- | --- |
| Petersen Criteria[^23^](file:///C:\Users\nam350\OneDrive%20-%20Newcastle%20University\Andrea%20McGrattan\Systematic%20Reviews\MCI%20SR_Bloss\Manuscript\Revision\Reviewed\Final\Co-Author%20review\Alz%20and%20Dementia\To%20SUBMIT\MCI%20conversion%20SR_Transfer_R1.docx#_ENREF_23)^,^ [^35^](file:///C:\Users\nam350\OneDrive%20-%20Newcastle%20University\Andrea%20McGrattan\Systematic%20Reviews\MCI%20SR_Bloss\Manuscript\Revision\Reviewed\Final\Co-Author%20review\Alz%20and%20Dementia\To%20SUBMIT\MCI%20conversion%20SR_Transfer_R1.docx#_ENREF_35)^,^ [^54^](file:///C:\Users\nam350\OneDrive%20-%20Newcastle%20University\Andrea%20McGrattan\Systematic%20Reviews\MCI%20SR_Bloss\Manuscript\Revision\Reviewed\Final\Co-Author%20review\Alz%20and%20Dementia\To%20SUBMIT\MCI%20conversion%20SR_Transfer_R1.docx#_ENREF_54)^,^ [^88^](file:///C:\Users\nam350\OneDrive%20-%20Newcastle%20University\Andrea%20McGrattan\Systematic%20Reviews\MCI%20SR_Bloss\Manuscript\Revision\Reviewed\Final\Co-Author%20review\Alz%20and%20Dementia\To%20SUBMIT\MCI%20conversion%20SR_Transfer_R1.docx#_ENREF_88) (n=5 studies) | | | | |
| Huang et al [^43^](file:///C:\Users\nam350\OneDrive%20-%20Newcastle%20University\Andrea%20McGrattan\Systematic%20Reviews\MCI%20SR_Bloss\Manuscript\Revision\Reviewed\Final\Co-Author%20review\Alz%20and%20Dementia\To%20SUBMIT\MCI%20conversion%20SR_Transfer_R1.docx#_ENREF_43) | China | Cox proportional hazard regression model | Age  Sex, education, memory complaints, stroke, CADL, CMMSE, FOM, RVR, BD, DS | Age (increasing)  Education, memory complaints, stroke, CADL, CMMSE, FOM, RVR, BD, DS |
| Li et al [^42^](file:///C:\Users\nam350\OneDrive%20-%20Newcastle%20University\Andrea%20McGrattan\Systematic%20Reviews\MCI%20SR_Bloss\Manuscript\Revision\Reviewed\Final\Co-Author%20review\Alz%20and%20Dementia\To%20SUBMIT\MCI%20conversion%20SR_Transfer_R1.docx#_ENREF_42) | China | Cox proportional hazard regression model | Sex, age, education, occupation, APOE e4, depression, MMSE, ADL,VRF (combined), hypertension, diabetes, hypercholesterolemia, obesity, myocardial infarction, atrial fibrillation, CVD, current smoking, daily drinking, treatment of VRF (some treated and all treated), and treatment of individual conditions including hypertension, diabetes, hypercholesterolemia, obesity, myocardial infarction, CVD | Sex (female), age (increasing), education (lower), occupation, APOE e4, smoking, alcohol consumption, VRF (combined), hypertension, diabetes, hypercholesterolemia, CVD, treatment of VRF (some and all treated) and treatment for hypertension, diabetes, hypercholesterolemia |
| Ding et al [^48^](file:///C:\Users\nam350\OneDrive%20-%20Newcastle%20University\Andrea%20McGrattan\Systematic%20Reviews\MCI%20SR_Bloss\Manuscript\Revision\Reviewed\Final\Co-Author%20review\Alz%20and%20Dementia\To%20SUBMIT\MCI%20conversion%20SR_Transfer_R1.docx#_ENREF_48) | China | Cox proportional hazard regression model | Sex, age, education, hypertension, diabetes, stroke, heart disease, smoking, drinking, APOE e4, MMSE | Age (increasing), MMSE (lower), APOE e4 |
| Yang et al [^47^](file:///C:\Users\nam350\OneDrive%20-%20Newcastle%20University\Andrea%20McGrattan\Systematic%20Reviews\MCI%20SR_Bloss\Manuscript\Revision\Reviewed\Final\Co-Author%20review\Alz%20and%20Dementia\To%20SUBMIT\MCI%20conversion%20SR_Transfer_R1.docx#_ENREF_47) | China | Logistic regression | Sex, age, aberrant motor behaviour, apathy/indifference, education, baseline MMSE, depression | Age (increasing), Baseline MMSE, depression |
| Yu et al [^45^](file:///C:\Users\nam350\OneDrive%20-%20Newcastle%20University\Andrea%20McGrattan\Systematic%20Reviews\MCI%20SR_Bloss\Manuscript\Revision\Reviewed\Final\Co-Author%20review\Alz%20and%20Dementia\To%20SUBMIT\MCI%20conversion%20SR_Transfer_R1.docx#_ENREF_45) | China | Multi State Model | Sex, age, education, hypertension, diabetes, reading, APOE e4 | Sex (female), age (increasing), hypertension, diabetes, APOE e4 |
| CIND based on Levy & Working Party of the International Psychogeriatric Association in collaboration with the WHO[^57^](file:///C:\Users\nam350\OneDrive%20-%20Newcastle%20University\Andrea%20McGrattan\Systematic%20Reviews\MCI%20SR_Bloss\Manuscript\Revision\Reviewed\Final\Co-Author%20review\Alz%20and%20Dementia\To%20SUBMIT\MCI%20conversion%20SR_Transfer_R1.docx#_ENREF_57) (n=1 study) | | | | |
| Baiyewu et al [^51^](file:///C:\Users\nam350\OneDrive%20-%20Newcastle%20University\Andrea%20McGrattan\Systematic%20Reviews\MCI%20SR_Bloss\Manuscript\Revision\Reviewed\Final\Co-Author%20review\Alz%20and%20Dementia\To%20SUBMIT\MCI%20conversion%20SR_Transfer_R1.docx#_ENREF_51) | Nigeria | Logistic Regression | Age, sex, depression, anxiety, alcohol consumption, smoking status, rural residence to age 19, education, hypertension, Parkinson’s disease, stroke, and APOE e4 | Age (increasing), sex (female) |
| Clinical Dementia Rating[^89^](file:///C:\Users\nam350\OneDrive%20-%20Newcastle%20University\Andrea%20McGrattan\Systematic%20Reviews\MCI%20SR_Bloss\Manuscript\Revision\Reviewed\Final\Co-Author%20review\Alz%20and%20Dementia\To%20SUBMIT\MCI%20conversion%20SR_Transfer_R1.docx#_ENREF_89) (n=1 study) | | | | |
| Montaño et al [^50^](file:///C:\Users\nam350\OneDrive%20-%20Newcastle%20University\Andrea%20McGrattan\Systematic%20Reviews\MCI%20SR_Bloss\Manuscript\Revision\Reviewed\Final\Co-Author%20review\Alz%20and%20Dementia\To%20SUBMIT\MCI%20conversion%20SR_Transfer_R1.docx#_ENREF_50) | Brazil | Poisson regression | Sex, age, education, ADL, MHSQ, vascular risk NPB, CDR, CDR-SB, cure rate | CDR |
| International Working Group (IWG)[^24^](file:///C:\Users\nam350\OneDrive%20-%20Newcastle%20University\Andrea%20McGrattan\Systematic%20Reviews\MCI%20SR_Bloss\Manuscript\Revision\Reviewed\Final\Co-Author%20review\Alz%20and%20Dementia\To%20SUBMIT\MCI%20conversion%20SR_Transfer_R1.docx#_ENREF_24) (n=1 study) | | | | |
| Paddick et al [^52^](file:///C:\Users\nam350\OneDrive%20-%20Newcastle%20University\Andrea%20McGrattan\Systematic%20Reviews\MCI%20SR_Bloss\Manuscript\Revision\Reviewed\Final\Co-Author%20review\Alz%20and%20Dementia\To%20SUBMIT\MCI%20conversion%20SR_Transfer_R1.docx#_ENREF_52) | Tanzania | Nonparametric tests: Mann-Whitney test and Chi-Squared | Sex, age, education, BMI, blood pressure, pulse pressure, hypertension, stroke, malaria, NPI |  |
| DSM-IV criteria[^38^](file:///C:\Users\nam350\OneDrive%20-%20Newcastle%20University\Andrea%20McGrattan\Systematic%20Reviews\MCI%20SR_Bloss\Manuscript\Revision\Reviewed\Final\Co-Author%20review\Alz%20and%20Dementia\To%20SUBMIT\MCI%20conversion%20SR_Transfer_R1.docx#_ENREF_38) (n=1 study) | | | | |
| Wang et al [^46^](file:///C:\Users\nam350\OneDrive%20-%20Newcastle%20University\Andrea%20McGrattan\Systematic%20Reviews\MCI%20SR_Bloss\Manuscript\Revision\Reviewed\Final\Co-Author%20review\Alz%20and%20Dementia\To%20SUBMIT\MCI%20conversion%20SR_Transfer_R1.docx#_ENREF_46) | China | Cox proportional hazard regression model | Sex, age, marital status, occupation, income, education, living area, subjective and objective memory impairment, memory decline, MMSE, hypertension, hyperlipidaemia, diabetes, exercise, community activities, reading, drinking, appetite, anxiety and fear | Age (increasing), hypertension, hyperlipidaemia, diabetes, exercise (low), anxiety, fear, APOE e4 |
| Yu et al [^44^](file:///C:\Users\nam350\OneDrive%20-%20Newcastle%20University\Andrea%20McGrattan\Systematic%20Reviews\MCI%20SR_Bloss\Manuscript\Revision\Reviewed\Final\Co-Author%20review\Alz%20and%20Dementia\To%20SUBMIT\MCI%20conversion%20SR_Transfer_R1.docx#_ENREF_44) | China | n/r | n/r | n/r |
| Dubois and Albert, 2004[^56^](file:///C:\Users\nam350\OneDrive%20-%20Newcastle%20University\Andrea%20McGrattan\Systematic%20Reviews\MCI%20SR_Bloss\Manuscript\Revision\Reviewed\Final\Co-Author%20review\Alz%20and%20Dementia\To%20SUBMIT\MCI%20conversion%20SR_Transfer_R1.docx#_ENREF_56) (n=1 study) | | | | |
| Godinho et al [^49^](file:///C:\Users\nam350\OneDrive%20-%20Newcastle%20University\Andrea%20McGrattan\Systematic%20Reviews\MCI%20SR_Bloss\Manuscript\Revision\Reviewed\Final\Co-Author%20review\Alz%20and%20Dementia\To%20SUBMIT\MCI%20conversion%20SR_Transfer_R1.docx#_ENREF_49) | Brasil | Cox proportional hazard regression model | Sex, age, education, family income, SRQ, MADRS, MMSE, depression, MCI (MCI vs cognitively normal), minor clinical illness, MMSE | MCI, MMSE |

**Key: ADL** Activities of Daily Living; **APOE e4** Apolipoprotein ε4 allele; **BD** Block Design; **BMI** Body Mass Index; **CADL** Chinese version of Activities of Daily Living; **CDR** Clinical Dementia Rating; **CDR-SB** Clinical Dementia Rating – Sum Box; **CMMSE** Chinese version of MMSE; **CVD** Cerebrovascular Disease; **DS** Digit Span; **FOM** Fuld Object-Memory Evaluation; **GDS** Geriatric Depression Scale; **MCI** Mild Cognitive Impairment; **MHSQ** Mental Health Screening Questionnaire; **MMSE** Mini Mental State Examination; **NPB** Neuropsychological Battery; **NPI** Neuropsychiatric Inventory; **n/r** Not reported; **RVR** Rapid Verbal Retrieval; **VRF** Vascular Risk Factors including a combination of hypertension, diabetes, hypercholesterolemia, obesity, myocardial infarction, CVD; **WHO** World Health Organisation.

**Supplementary Material Table 3** Quality of studies according to the Newcastle Ottawa Scale [^40^](file:///C:\Users\nam350\OneDrive%20-%20Newcastle%20University\Andrea%20McGrattan\Systematic%20Reviews\MCI%20SR_Bloss\Manuscript\Revision\Reviewed\Final\Co-Author%20review\Alz%20and%20Dementia\To%20SUBMIT\MCI%20conversion%20SR_Transfer_R1.docx#_ENREF_40)

|  | Selection | | | | Comparability | Outcome | | | Total Score (out of 8) |
| --- | --- | --- | --- | --- | --- | --- | --- | --- | --- |
| Author | Representativeness of the exposed cohort | Selection of the non exposed cohort | Ascertainment of exposure | Demonstration that outcome of interest was not present at start of study | Comparability of cohorts on the basis of the design or analysis | Assessment of outcome | Was follow-up long enough for outcomes to occur | Adequacy of follow up of cohorts |  |
| Huang et al [^43^](file:///C:\Users\nam350\OneDrive%20-%20Newcastle%20University\Andrea%20McGrattan\Systematic%20Reviews\MCI%20SR_Bloss\Manuscript\Revision\Reviewed\Final\Co-Author%20review\Alz%20and%20Dementia\To%20SUBMIT\MCI%20conversion%20SR_Transfer_R1.docx#_ENREF_43) | *****  Somewhat as random sampling used; not nationally representative | *****  Somewhat as random sampling used; not nationally representative | *****  Validated tool | *****  Only MCI selected | *****  Age, Sex, Education | *****  Validated tool for dementia used | *****  >1 year | *  Response Rate <75%, however stated that no differences between participants & those lost to follow up | 8 |
| Li et al [^42^](file:///C:\Users\nam350\OneDrive%20-%20Newcastle%20University\Andrea%20McGrattan\Systematic%20Reviews\MCI%20SR_Bloss\Manuscript\Revision\Reviewed\Final\Co-Author%20review\Alz%20and%20Dementia\To%20SUBMIT\MCI%20conversion%20SR_Transfer_R1.docx#_ENREF_42) | *****  Somewhat as random sampling used; not nationally representative | *****  Somewhat as random sampling used; not nationally representative | *****  Validated tool | *****  Only MCI selected | *****  Age, Sex, Education | *****  Validated tool for dementia used | *****  >1 year | *****  Response rate >75% | 8 |
| Ding et al[^48^](file:///C:\Users\nam350\OneDrive%20-%20Newcastle%20University\Andrea%20McGrattan\Systematic%20Reviews\MCI%20SR_Bloss\Manuscript\Revision\Reviewed\Final\Co-Author%20review\Alz%20and%20Dementia\To%20SUBMIT\MCI%20conversion%20SR_Transfer_R1.docx#_ENREF_48) | Does not indicate if random selection was used and cannot confirm if representative | Does not indicate if random selection was used and cannot confirm if representative | *****  Validated tool | *****  Only MCI selected | *****  Age, Sex, Education | *****  Validated tool for dementia used | *****  >1 year | *****  Response rate >75% | 6 |
| Yang et al[^47^](file:///C:\Users\nam350\OneDrive%20-%20Newcastle%20University\Andrea%20McGrattan\Systematic%20Reviews\MCI%20SR_Bloss\Manuscript\Revision\Reviewed\Final\Co-Author%20review\Alz%20and%20Dementia\To%20SUBMIT\MCI%20conversion%20SR_Transfer_R1.docx#_ENREF_47) | Not all participants included in the study were selected at random | Not all participants included in the study were selected at random | *****  Validated tool | *****  Only MCI selected | *****  Age, Sex, Education | *****  Validated tool for dementia used | *****  >1 year | Response rate was 71% | 5 |
| Yu et al[^45^](file:///C:\Users\nam350\OneDrive%20-%20Newcastle%20University\Andrea%20McGrattan\Systematic%20Reviews\MCI%20SR_Bloss\Manuscript\Revision\Reviewed\Final\Co-Author%20review\Alz%20and%20Dementia\To%20SUBMIT\MCI%20conversion%20SR_Transfer_R1.docx#_ENREF_45) | *****  Somewhat as random sampling used; not nationally representative | *****  Somewhat as random sampling used; not nationally representative | *****  Validated tool | *****  Only MCI selected | *****  Age, Sex, Education | *****  Validated tool for dementia used | *****  >1 year | *****  Response rate >75% | 8 |
| Baiyewu et al [^51^](file:///C:\Users\nam350\OneDrive%20-%20Newcastle%20University\Andrea%20McGrattan\Systematic%20Reviews\MCI%20SR_Bloss\Manuscript\Revision\Reviewed\Final\Co-Author%20review\Alz%20and%20Dementia\To%20SUBMIT\MCI%20conversion%20SR_Transfer_R1.docx#_ENREF_51) | *****  Somewhat as random sampling used; not nationally representative | *****  Somewhat as random sampling used; not nationally representative | *****  Validated tool | *****  Only MCI selected | *****  Age, Sex, Education | *****  Validated tool for dementia used | *****  >1 year | *****  Response rate >75% | 8 |
| Montano et al [^50^](file:///C:\Users\nam350\OneDrive%20-%20Newcastle%20University\Andrea%20McGrattan\Systematic%20Reviews\MCI%20SR_Bloss\Manuscript\Revision\Reviewed\Final\Co-Author%20review\Alz%20and%20Dementia\To%20SUBMIT\MCI%20conversion%20SR_Transfer_R1.docx#_ENREF_50) | *****  Somewhat as random sampling used; not nationally representative | *****  Somewhat as random sampling used; not nationally representative | *****  Validated tool | *****  Only MCI selected | *****  Age, Sex, Education | *****  Validated tool for dementia used | *****  >1 year | *  The response rate was less than 75% however stated that no differences between participants & those lost to follow up | 8 |
| Paddick et al[^52^](file:///C:\Users\nam350\OneDrive%20-%20Newcastle%20University\Andrea%20McGrattan\Systematic%20Reviews\MCI%20SR_Bloss\Manuscript\Revision\Reviewed\Final\Co-Author%20review\Alz%20and%20Dementia\To%20SUBMIT\MCI%20conversion%20SR_Transfer_R1.docx#_ENREF_52) | *****  Somewhat as random sampling used; not nationally representative | *****  Somewhat as random sampling used; not nationally representative | *****  Validated tool | *****  Only MCI selected | *****  Age, Sex, Education | *****  Validated tool for dementia used | *****  >1 year | *****  Response rate >75% | 8 |
| Wang et al[^46^](file:///C:\Users\nam350\OneDrive%20-%20Newcastle%20University\Andrea%20McGrattan\Systematic%20Reviews\MCI%20SR_Bloss\Manuscript\Revision\Reviewed\Final\Co-Author%20review\Alz%20and%20Dementia\To%20SUBMIT\MCI%20conversion%20SR_Transfer_R1.docx#_ENREF_46) | *****  Somewhat as random sampling used; not nationally representative | *****  Somewhat as random sampling used; not nationally representative | *****  Validated tool | *****  Only MCI selected | *****  Age, Sex, Education | *****  Validated tool for dementia used | *****  >1 year | *****  Response rate >75% | 8 |
| Yu et al[^44^](file:///C:\Users\nam350\OneDrive%20-%20Newcastle%20University\Andrea%20McGrattan\Systematic%20Reviews\MCI%20SR_Bloss\Manuscript\Revision\Reviewed\Final\Co-Author%20review\Alz%20and%20Dementia\To%20SUBMIT\MCI%20conversion%20SR_Transfer_R1.docx#_ENREF_44) | Does not indicate if random selection was used and cannot confirm if representative | Does not indicate if random selection was used and cannot confirm if representative | *****  Validated tool | *****  Only MCI selected | Not reported | *****  Validated tool for dementia used | *****  >1 year | *****  Response rate >75% | 5 |
| Godhino et al [^49^](file:///C:\Users\nam350\OneDrive%20-%20Newcastle%20University\Andrea%20McGrattan\Systematic%20Reviews\MCI%20SR_Bloss\Manuscript\Revision\Reviewed\Final\Co-Author%20review\Alz%20and%20Dementia\To%20SUBMIT\MCI%20conversion%20SR_Transfer_R1.docx#_ENREF_49) | *****  Sample from a nationally representative / random sampling used | *****  Sample from a nationally representative / random sampling used | *****  Validated tool | *****  Only MCI selected | *****  Age, Sex, Education | *****  Validated tool for dementia used | *****  >1 year | *****  Response rate >75% | 8 |

**Supplementary Material: Table 4: Details of conversion rate method for studies included in Meta-Analysis**

| **No** | **Study (Year)** | **Conversion rate method** |
| --- | --- | --- |
| 1 | Huang(2005) | Conversion rate was calculated based on number of person-years of follow-up. |
| 2 | Li(2011) | Not clear (*denoted in percentage of those from MCI to AD per year*). |
| 3 | Yang(2020) | Not mentioned. |
| 4 | Yu(2013) | Transition probability using Multi-State Markov Model. |
| 5 | Paddick(2015) | Not clear (*denoted in percentage of those from MCI to AD over 4 year period*). |
| 6 | Godinho(2012) | Quotient of the percentage of MCI subjects who converted to probable AD and follow-up and the mean delay to follow-up. |
